# Adult-Onset Reye's Syndrome and the Risks of Low-Dose Aspirin Rechallenge: A Review of Current Literature and Future Directions

**DOI:** 10.7759/cureus.96332

**Published:** 2025-11-07

**Authors:** Christopher Gibson, James Noyes, Myles Goliger, Junjing Guo

**Affiliations:** 1 Medicine, SUNY Downstate Health Science University, Brooklyn, USA; 2 Hematology and Medical Oncology, SUNY Downstate Health Science University, Brooklyn, USA

**Keywords:** adult-onset, aspirin rechallenge, atherosclerosis, reye’s syndrome, thrombosis

## Abstract

Reye’s syndrome is a rare encephalopathy with liver involvement correlated with salicylate drug use in the setting of many viral infections, especially influenza B and varicella, which are disproportionately prevalent in pediatric patients. Reye’s syndrome has declined markedly since the 1960s due to public health campaigns and the adoption of alternative antipyretics. The risks associated with salicylate use in adult patients who survived childhood Reye’s syndrome are unknown. We review the current literature regarding the prevalence of adult-onset Reye’s syndrome, cases of recurrence, and low-dose aspirin rechallenge in a patient who reported a childhood history of Reye’s syndrome. Much of the available literature on Reye’s syndrome in adults is dated, and a confirmatory diagnosis in many of these cases is deemed controversial, as certain diagnostic tests were not performed. The publication of additional rechallenge trials that include documentation of associated symptoms and lab work, as well as confirmation of childhood disease, would greatly facilitate the management of adult patients with a childhood history of Reye’s syndrome by providing a confident estimation of the risk associated with low-dose aspirin rechallenge. Given the lack of sufficient data on the risk associated with low-dose aspirin rechallenge, careful evaluation and further research are necessary to define safe protocols for salicylate drug use in adults with a history of Reye’s syndrome.

## Introduction and background

Clinical significance

Reye’s syndrome is a rare noninflammatory encephalopathy of unknown etiology with liver manifestations that is associated with the use of salicylate drugs in the setting of certain viral infections [1]. It is most commonly seen in children who take aspirin (or other salicylate-containing medications) while infected with viruses, especially varicella or influenza B. The onset of symptoms of Reye’s syndrome is typically three to five days after infection [2] and is characterized by conscious lethargy and irritability with vomiting. The illness can then progress to neurologic symptoms, including coma and flaccid paralysis. Hepatopathy is suggested by markedly high aspartate aminotransferase (AST) and alanine aminotransferase (ALT) values, which are at least three times greater than normal, as well as hyperbilirubinemia in some patients. The increase in intracranial pressure due to cerebral edema seen in the syndrome can restrict cerebral blood flow and result in brain damage or death [3]*.*

Historical context

One of the most consistent findings in the illness is an increase in blood ammonia, of which peak levels appear to be of prognostic value early in the course of the encephalopathy [4]*.* Indeed, in a case of Reye’s syndrome in one of the oldest patients ever reported, a 59-year-old woman woke up 48 hours after her intubation, exhibiting an improvement in mental state that corresponded directly with a fall in the plasma ammonia level [5]*.* Therefore, since Reye’s syndrome can go unrecognized, the importance of measuring plasma ammonia in cases of acute encephalopathy with profuse vomiting has been emphasized [6]*,* although more detailed analyses of hepatic enzymes and cytologic abnormalities of hepatic tissue specimens also have diagnostic value [5]. 

Patients with certain inborn errors of metabolism, such as medium-chain acyl-CoA dehydrogenase deficiency, can also develop metabolic disorders, especially of fatty acid oxidation, that lead to different forms of Reye's-like disease, and have historically been misdiagnosed as such [7]*.* Therefore, central to current theory regarding the pathophysiology of Reye’s syndrome is the involvement of mitochondrial injury that is secondary to disordered metabolism, as many mitochondrial enzymes are decreased in its setting [3].

Knowledge gap and potential mechanisms involving salicylates

While the etiology of this illness is not well understood, it is known that salicylate and its metabolites (i.e., hydroxyhippurate and gentisate) directly inhibit long-chain-3-hydroxyacyl-CoA dehydrogenase, disrupting Q-oxidation [8]*.* A similar metabolic deficiency could be responsible for the intrahepatic accumulation of unusual short, medium, and long-chain acyl-CoA intermediates (esters) of fatty acid and branched-chain amino acid oxidation that has been observed in Reye’s syndrome. This impaired fatty acid oxidation in mitochondria can lead to the accumulation of hepatocellular lipid droplets, also known as steatosis, which is the key hepatic hallmark of the syndrome [9]*.* Furthermore, these acyl-CoA intermediates deplete mitochondrial ATP, suggesting that the hyperammonemia observed in Reye’s syndrome [10,3,11] is attributable to the inhibition of ureagenesis, a process that requires mitochondrial ATP [9]*.* The accumulation of high concentrations of ammonia leads to encephalopathy and anicteric hepatitis (with a three-fold increase in liver enzymes) that collectively represent the hallmark clinical pattern of Reye’s syndrome [12]. 

Purpose of review

With respect to the observed decline of Reye's syndrome over the last few decades, recent literature data reveal that this is related to more accurate modern diagnosis of infectious, metabolic, or toxic disease. Furthermore, several epidemiological studies have provided evidence that not only the use of acetylsalicylic acid but also that of phenothiazines and other antiemetics is significantly greater in Reye's syndrome cases than in controls. The information obtained from these studies raises questions concerning the proposed link between acetylsalicylic acid and Reye's syndrome [13]. Nevertheless, the risks associated with salicylate use in adult patients who survived an accurately diagnosed episode of childhood Reye’s syndrome are unknown. This review examines the current literature regarding the rates of adult-onset Reye's syndrome, the chances of recurrence of childhood Reye's syndrome, and the risk of rechallenge with low-dose aspirin in adult patients who had childhood Reye's syndrome. The purpose of this review is to equip physicians with information to help them assess the risk of developing the syndrome linked to low-dose aspirin use, specifically to prevent thrombotic or atherosclerotic events, as well as the risk associated with accidentally taking salicylate-containing drugs, particularly those sold over the counter.

## Review

Methods

We performed a narrative review of the literature on Reye's syndrome with or without salicylates as a risk factor. We performed multiple searches using the PubMed (NIH) database (1960-2025). The search was limited to human studies published in English. Keyword combinations of the medical subject headings (MeSH) included ‘‘Reye's", ‘‘Reye's syndrome",’ “aspirin Reye's”, ‘‘aspirin Reye's syndrome’’, ‘‘Reye's syndrome aspirin’,’ "salicylates Reye's", salicylates Reye's syndrome," "Reye's syndrome salicylates" and all of these search terms prefaced by the term "adult." Case report extraction fields were obtained by selecting "Case Reports" under article type. We conducted secondary searches by reviewing the references of primary articles to identify additional articles for inclusion and critical review. For the discussion of the risk of adult Reye's syndrome in patients taking salicylates, we included all studies of Reye's or Reye's-like syndrome in adults. We excluded studies published before 1960 when Reye's syndrome had not yet been hypothesized as a clinical entity.

No quantitative synthesis was performed, and a statistical review was not necessary since the analyses did not include pooled or comparative data.

Rationale for assessing the risk of Reye's syndrome in adults

Reye’s syndrome was first recognized in 1963 and was studied in great depth from the 1960s through the 1980s [14]. Such studies highlighted the gravity of the syndrome, with most studies showing a roughly 30-40% death rate in affected children and a roughly 10% incidence of neurological damage [1]. These reported high rates of mortality and morbidity provoked a national effort by health officials in the United States and other developed countries to discourage the use of aspirin in children fighting infections, with parents advised to give their children acetaminophen instead of aspirin as needed for discomfort and fever when fighting viral infections [15]. Although a recent study reported a fatality in a 12-year-old Reye's syndrome patient with salicylate intake [16], several recent cases of Reye's syndrome in children were not associated with salicylate use [17].

These efforts have driven down the incidence of new cases of Reye’s syndrome considerably over recent decades [18]. Nevertheless, the thousands of survivors with confirmed cases of childhood Reye’s syndrome in the past six decades in the US alone [6] are left with unanswered questions regarding their prospective use of salicylates in the setting of viral infections and their risk of recurrence.

An analysis of 186 reports of Reye's syndrome obtained from the US Food and Drug Administration Adverse Event Reporting System and the Japanese Adverse Drug Event Report databases concluded that the possible risk of the disease occurring in adults, females in particular, should be considered [19]. In the adult population, low-dose aspirin has been a standard oral treatment of coronary artery disease for recurrent myocardial infarction prophylaxis [20], treatment of deep vein thrombosis, and prevention of pulmonary embolism [21], ischemic stroke [22], or transient ischemic attack [23]. Also, low-dose aspirin usage is associated with a reduced risk of bleeding compared to other treatments such as oral apixaban [24] and combinations of aspirin with other medications for thrombotic or atherosclerotic diseases [20], which makes it a safe alternative for certain patients with specific risk factors.

Aside from environmental and lifestyle factors, advancing age is associated with an increased risk of developing such cardiovascular issues, particularly in individuals above the age of 45 [25]. Considering the popularity of low-dose aspirin for preventing thrombotic or ischemic events in this demographic, it is important to determine the risk associated with taking salicylate-containing medications for those who have survived Reye’s syndrome, as current guidelines are unclear. Understanding this risk will better inform the management of adult patients with a childhood history of Reye’s syndrome who have thrombotic or atherosclerotic diseases and who do not respond to or cannot tolerate other oral therapies or injections, as well as patients who are taking salicylates for other indications. These include non-aspirin medications that many patients may not be aware contain salicylates, such as bismuth subsalicylate, a common over-the-counter remedy for gastrointestinal upset, and some ulcerative colitis medications, such as mesalamine.

Reye's syndrome in adults

In its presentation, diagnosis, and management, adult-onset Reye's syndrome is similar in most respects to that seen in pediatric populations, with comparable general, gastrointestinal, hepatic, and intracranial symptoms [4]. What separates the two is most clearly seen in confirmatory diagnostic and treatment modalities. First, obtaining a liver biopsy early on in the course of the illness may assist in excluding alternative hepatic pathologies, as Reye’s syndrome typically shows microvesicular steatosis without necrosis or inflammation. Additionally, a computed tomography scan of the brain can be used to evaluate intracranial edema. The treatment of this encephalopathy in adults to reduce intracranial pressure is emphasized more so than in children, since hepatic dysfunction tends to be less fulminant in adults, so encephalopathy predominates clinically. Encephalopathy is the most life-threatening complication of the disease, increasing the risk of rapid neurological impairment, whereas hepatic involvement progresses more slowly.

Diagnosis of adult Reye's syndrome is more difficult than in pediatric cases, and its prevalence is also much lower in adults than in children [26]. Determining the current rates of adult-onset Reye's syndrome is deeply complicated by the contradictory figures published in past years, the many cases that have been attributed to other pathologies, and the lack of recent publications on adult Reye's syndrome. The most recent study of adult Reye's syndrome cases since the illness was first described in 1963, published in 1991, listed 26 reported cases [27].

Despite these challenges, the rare cases of adult Reye's syndrome have been reported primarily in patients younger than 35 [28]. As of 1986, the oldest patient reported with a confirmed case of Reye's syndrome was 61 years old, presenting in the typical manner after taking aspirin 600 mg every 8-10 hours for his symptoms in addition to aspirin 300 mg, which he took daily for stroke prevention [28]. There was a second case reported for a 61-year-old patient with multiple acyl-coenzyme A dehydrogenase deficiency who presented with a Reye-like syndrome after taking aspirin for coronary artery disease [29]. 

Recurrence of Reye's syndrome

There is little information regarding reported cases of recurrent Reye's syndrome in childhood; there are only two suggested instances where recurrence may have occurred, one of which was published in 1978 and described the first documented case of recurrence [30]. This case involved a three-year-old girl who had classic Reye's syndrome symptoms following salicylate use with a presumed influenza B infection, which occurred around one year after a confirmed case of Reye's syndrome. The authors of this case report, though, have received criticism as influenza B was never confirmed in this patient, and appropriate serological tests were not performed to rule out other causes for the observed encephalopathy [31]. This study highlights the need for confirmatory diagnosis modalities in suspected cases of recurrence to ensure accuracy in the documentation of such perceived anomalies. Since this article was published, there have not been any publications of such cases. However, this is likely a result of the sharply decreased rates of Reye's syndrome due to efforts to bring awareness to the general public of the risks associated with aspirin use for the treatment of childhood viral infections.

Risk of rechallenge of low-dose aspirin in adult patients who had childhood Reye's syndrome

The risk of recurrence of Reye's syndrome in an adult who had the illness during childhood is not well understood, as such cases have not been published. Additionally, aspirin rechallenge has only been tried recently in one adult patient. This was described in a case report from 2019 showing the results of low-dose aspirin in a patient who had survived childhood Reye's syndrome 30 years earlier, after resuscitation from cardiac arrest due to a 99% proximal left anterior descending coronary artery occlusion [32]. In the hospital, he was given eptifibatide in substitution for aspirin, as aspirin was listed as an allergy in his medical record.

The patient reported having Reye's syndrome as a child, which prompted him to list aspirin as an allergy. In a later discussion with the patient and his mother regarding his history, specifically his history of Reye's syndrome, it was revealed that he suffered an unspecified acute infection with fever at 12 years old. After the infection resolved, he experienced short-term memory loss and lost his ability to speak. The dose of aspirin given during the infection was unknown, and the hospital did not obtain salicylate levels, nor was there a confirmatory liver biopsy performed. A true allergy to aspirin with throat swelling was also admitted during the interview.

Despite this history, the patient agreed to rechallenge with aspirin and was discharged with instructions to take aspirin 25 mg daily. Liver function tests (AST and ALT) were slightly elevated on day 0 (55 and 56 U/L, respectively), after which both markers remained normalized throughout the retrial, along with the rest of the recorded metabolic panel.

Concerning the risk associated with developing Reye's syndrome in adult patients, its prevalence in such populations suggests a low overall risk of taking salicylate-containing drugs, especially considering how many adult patients take low-dose aspirin for prophylaxis [14]. Although current data is not available, with such few reported cases, due largely to efforts to reduce aspirin use in the presence of viral infections through substitution with acetaminophen, the current number of new cases of adult Reye's syndrome is unlikely to be significantly greater than those reported in 1991 [15]. This also provides context for the lack of publications on adult Reye's syndrome over the past few decades. Another possible factor is the weaker association between salicylates and adult Reye's syndrome compared to children, which has been reported in some studies [26,28].

The specific age demographics in reported adult Reye's syndrome cases (rarely above 35 years old) and the context of the 61-year-old patient’s relatively high aspirin dosage (1500 - 2100 mg per day) help provide insight into the relative risk of prescribing low-dose aspirin to patients above 45 years old [28]. It is also important to note, however, especially concerning this case, that the etiologic relationship between salicylate dose and adult Reye's syndrome occurrence has yet to be established.

Assessing risk based on prevalence in adult patients is complicated by the lack of data concerning recurrence and subsequent risk in adult patients with a childhood history of Reye's syndrome, particularly in patients who do not have inborn errors of metabolism. This complication is due, in part, to the unknown etiology of Reye's syndrome. Furthermore, this also creates hurdles in developing concrete diagnostic criteria, which further complicates the interpretation of published data. However, since the vast majority of Reye's syndrome cases are seen in younger adults, less than 35 years of age [28], and the prevalence of the syndrome is much higher in children than adults, there must be some etiological factor that disproportionately puts younger patients at risk. Without a complete understanding of the etiology of Reye's syndrome, patients cannot be given a true estimation of the associated risk when considering an aspirin rechallenge.

Such a rechallenge was conducted with low-dose aspirin in a patient following a myocardial infarction and stent placement [32]. However, this case report does not have confirmed evidence of a prior case of Reye's syndrome in the form of hospital records of lab work or biopsies, nor measurements of serum salicylate levels. Therefore, it must be considered that this aspirin rechallenge was conducted in a patient suspected to have a history of Reye's syndrome, the prior diagnosis of which was based only on symptoms. The diagnosis of Reye's in the rechallenge adult case was also made without any confirmation from biopsy results, serum salicylate levels, elevated serum ammonia [4-6], or measurements of the short- and medium-chain fatty acids or their metabolites that have been found in the blood and urine of Reye’s patients [3,33-34]. Such short- and medium-chain fatty acids are the deacylated metabolites of unusual acyl-CoA-fatty acid esters that have been shown to accumulate in the liver in Reye’s syndrome [9]. Thus, a properly designed low-dose aspirin rechallenge trial that measures the most important diagnostic markers in an adult patient with a childhood history of Reye's syndrome has yet to be conducted. It is also recommended that the rechallenge trial include a therapeutic aspirin dosage for cardiovascular disease, such as 81 mg, for optimal clinical relevance. 

## Conclusions

Reye's syndrome is a rare, dangerous, and potentially deadly encephalopathy with hepatic involvement correlated with salicylate use in the setting of viral infections, especially influenza B and varicella infections. Management of adult patients with a childhood history of Reye's syndrome is unclear in that the risks of Reye's syndrome recurrence from taking salicylate-containing drugs are unknown in such patients. Determining the safety of low-dose aspirin for thrombotic or atherosclerotic prophylaxis among survivors of childhood Reye's syndrome could open up another oral route option for patients at risk for such events. This option has been a hallmark of such prophylactic treatments of cardiovascular diseases for decades. There is currently very little published literature regarding the risks associated with a recurrence of Reye's syndrome, and there are no studies that examine the safety of low-dose aspirin rechallenge in confirmed cases of Reye's syndrome. Thus, there is a need to conduct and report rechallenges in such confirmed cases with documentation of liver function tests, serum salicylate levels, serum ammonia, blood and urine levels of short- and medium-chain fatty acids, and associated symptoms, either through controlled trials or by combining results of several case series via meta-analysis. It is also recommended that all children with manifestations suggestive of Reye's syndrome be tested for inherent errors of metabolism (IEM). Early recognition and treatment of Reye's and Reye's-like syndromes, including presumptive treatment for possible IEM, are essential to prevent related mortality and optimize the likelihood of recovery without neurologic impairment.
